# P-1761. The Host Transcriptional Response to Cryptococcal Infection Reflects Dysregulated Innate and Adaptive Immune System Functions that Increases Vulnerability to Infection

**DOI:** 10.1093/ofid/ofaf695.1932

**Published:** 2026-01-11

**Authors:** Julie M Steinbrink, Beatrice Sim, Cameron Miller, Deng B Madut, John A Crump, Venance Maro, Matthew Rubach, Micah T McClain

**Affiliations:** Duke University Medical Center, Durham, NC; Duke University Medical Center, Durham, NC; Duke University Medical Center, Durham, NC; Duke University, Durham, North Carolina; Duke University Medical Center, Duke Global Health Institute, Durham, North Carolina; Kilimanjaro Christian Medical Centre, Tumaini University, Moshi, Kilimanjaro, Tanzania; Duke University, Durham, North Carolina; Duke University, Durham, North Carolina

## Abstract

**Background:**

*Cryptococcus* species are opportunistic fungal pathogens that cause life-threatening infections; however, clinical presentation often overlaps with other febrile syndromes, complicating diagnosis. We leveraged whole-blood transcriptomic analysis to define unique, conserved elements of the immune response to real-world *Cryptococcus* infection to clarify elements of increased susceptibility as well as improve diagnosis.

**Figure:**
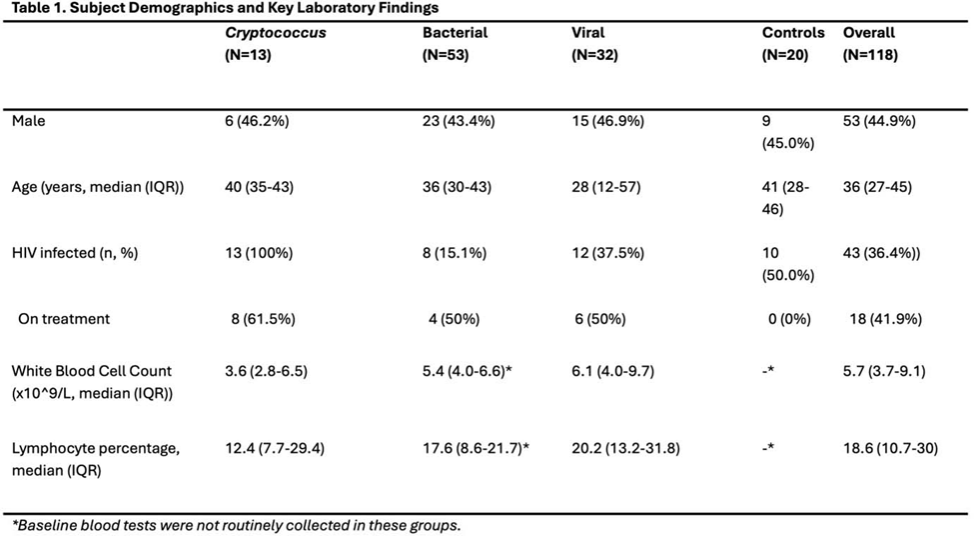

**Figure:**
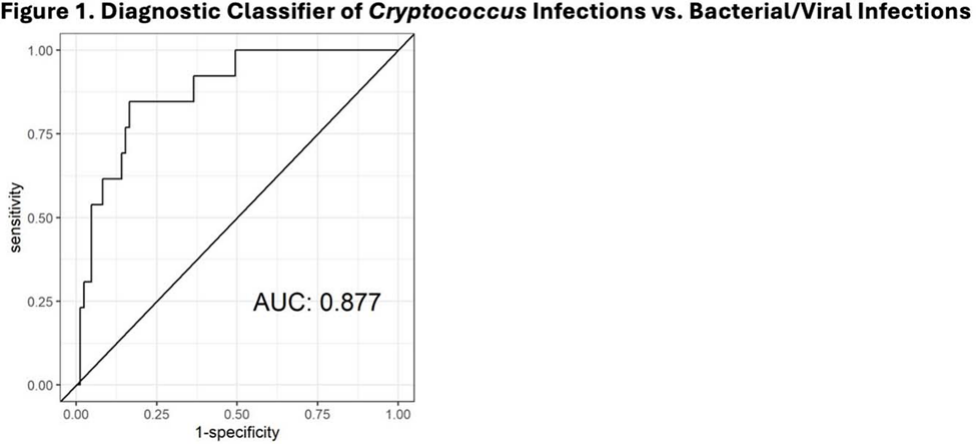

**Methods:**

Whole blood was collected by PAXgene for RNA sequencing from adult febrile inpatients at a Tanzanian medical center to compare gene expression between patients with cryptococcosis (defined as positive blood cultures or cryptococcal antigen >1:4), combined bacterial/viral pathogens, and healthy controls.

Differential expression and gene set enrichment analyses were used to characterize expression differences between pathogen-class groups. Penalized logistic regression was used to identify signatures capable of identifying *Cryptococcus* infection.

**Results:**

Overall, 118 patients were included (Table 1). 3,853 genes were upregulated in response to cryptococcosis compared to healthy subjects, which reflected complement activation, neutrophil function, type I interferon signaling, and myeloid dendritic cell differentiation. Pathways clustered around activation of innate and adaptive immune responses.

Univariate comparison of cryptococcal to combined bacterial/viral infection found that upregulated genes corresponded to components of innate, adaptive, and humoral immunity including type I interferon and tumor necrosis factor pathways. We identified a 28-gene classifier that distinguished between cryptococcal and combined bacterial/viral infections with an AUC of 0.877 (Figure 1).

**Conclusion:**

The human host immune response to cryptococcosis suggests that uniquely dysregulated elements - like elevated type I interferon - may lead to a more permissive state for infection (as suggested in animal models) and indicate a complex and context-dependent role for these pathways in human disease. Overall, the host response as manifested in transcriptional profiles informs our understanding of the pathophysiology of cryptococcal infection and demonstrates promise for the development of future novel diagnostic approaches.

**Disclosures:**

Julie M. Steinbrink, MD, MHS, Biomeme: patents for gene expression classifiers of fungal infection|McGraw Hill Publishing: royalties Micah T. McClain, MD, PhD, Biomeme: patents for gene expression classifiers of fungal infection|Darwin Biosciences: Board Member|UpToDate: Advisor/Consultant

